# Limitations of Augmentation Index in the Assessment of Wave Reflection in Normotensive Healthy Individuals

**DOI:** 10.1371/journal.pone.0059371

**Published:** 2013-03-27

**Authors:** Alun D. Hughes, Chloe Park, Justin Davies, Darrel Francis, Simon A. McG Thom, Jamil Mayet, Kim H. Parker

**Affiliations:** 1 International Centre for Circulatory Health, National Heart and Lung Institute, Imperial College London and Imperial College Healthcare NHS Trust, London, United Kingdom; 2 Department of Bioengineering, Imperial College London, United Kingdom; University Hospital of Würzburg, Germany

## Abstract

**Objectives:**

Augmentation index (AIx) is widely used as a measure of wave reflection. We compared the relationship between AIx and age, height and sex with ‘gold standard’ measures of wave reflection derived from measurements of pressure and flow to establish how well AIx measures wave reflection.

**Materials and Methods:**

Measurements of carotid pressure and flow velocity were made in the carotid artery of 65 healthy normotensive individuals (age 21–78 yr; 43 male) and pulse wave analysis, wave intensity analysis and wave separation was performed; waveforms were classified into type A, B or C. AIx, the time of the first shoulder (T_s_), wave reflection index (WRI) and the ratio of backward to forward pressure (P_b_/P_f_) were calculated.

**Results:**

AIx did not correlate with log WRI or P_b_/P_f_. When AIx was restricted to positive values AIx and log WRI were positively correlated (r = 0.33; p = 0.04). In contrast log WRI and P_b_/P_f_ were closely correlated (r = 0.66; p<0.001). There was no correlation between the T_s_ and the timing of P_b_ or the reflected wave identified by wave intensity analysis. Wave intensity analysis showed that the morphology of type C waveforms (negative AIx) was principally due to a forward travelling (re-reflected) decompression wave in mid-systole. AIx correlated positively with age, inversely with height and was higher in women. In contrast log WRI and P_b_/P_f_ showed negative associations with age, were unrelated to height and did not differ significantly by gender.

**Conclusions:**

AIx has serious limitations as a measure of wave reflection. Negative AIx values derived from Type C waves should not be used as estimates of wave reflection magnitude.

## Introduction

High blood pressure is a major cause of cardiovascular disease [1]. Wave reflection is an important determinant of systolic blood pressure and systolic hypertension [2], [3]; and is an independent predictor of cardiovascular risk in some studies [4]–[7]. Augmentation index (AIx), the pressure difference between the shoulder on the pressure wave and systolic pressure expressed as a ratio of pulse pressure is widely used as a proxy of wave reflection [2]. It has the advantage that it does not require concurrent measurement of blood flow; however the validity of AIx as a measure of reflection is uncertain as it is also influenced by pulse wave velocity and other factors. Recently it has also been suggested that AIx may be more indicative of arterial compliance and reservoir function than wave reflection [8].

The majority of studies using AIx as a measure of wave reflection have reported that AIx increases with increasing age [9], which has been interpreted as indicating that wave reflection increases with age [10], [11]. However some recent studies using other ‘gold standard’ measures of wave reflection have provided contradictory evidence regarding changes in wave reflection with ageing [3], [12]. AIx has also been reported to correlate inversely with height [11], [13], but there are no reports examining this relationship using wave separation techniques. AIx has also been found to be higher in women across the age range [11], [12], [14] and this gender difference is partly but not completely explained by differences in height [11], [14]. However studies using measures of wave reflection based on wave separation have not consistently found differences by gender [3], [12].

We hypothesized that these discrepancies could be due to limitations of AIx as a measure of wave reflection, particularly when type C waveforms are included in analyses (i.e. when AIx is negative). Therefore we compared AIx and measures of wave reflection based on pressure and flow in terms of the relationships with age, sex and height. In addition we used wave intensity analysis to determine the underlying wave patterns responsible for the different types of pressure waveform described in the literature to provide an explanation for the inconsistencies between AIx and other measures of wave reflection.

## Materials and Methods

### Participants

Healthy individuals of either sex, aged 21–78 years were recruited by advertising. Participants were excluded if they had any chronic disease, including known cardiovascular disease or hypertension, or if they were taking any medications with the exception of oral contraceptives. All studies were approved by the St Mary’s Hospital local research ethics committee and all participants gave written informed consent and all clinical investigation was conducted according to the principles expressed in the Declaration of Helsinki.

### Investigations

Participants were requested to refrain from smoking, alcohol or caffeine-containing beverages for 24 h prior to the study. All studies were conducted in a temperature-controlled darkened room, with subjects having rested supine for at least 10 minutes. Brachial BP was measured using a validated, semi-automated device (Omron 705CP, Omron) [15] after ≥5 minutes rest. The BP waveform was measured in the right common carotid artery by applanation tonometry using a Millar tonometer (SPT-301, Millar Instruments Inc, Houston, Tx, USA) and calibrated to brachial artery BP as previously described [10], [16]. Carotid pressure waveforms were monitored during acquisition to ensure high quality and stability of recordings over at least 1 minute of measurement. Flow velocity measurements were also made in the right common carotid artery by pulsed wave Doppler with an HDI 5000 ultrasound machine (Philips Medical Systems, Best, The Netherlands) equipped with a 7.5–10 MHz linear array transducer at a Doppler angle of 60° in a 1 mm sample volume placed in the centre of the vessel ∼2 cm from the carotid bulb. Pressure data were collected first, followed by the velocity. An ECG was also recorded to allow ensemble averaging of waveforms and to provide a fiducial point for timing events in the cardiac cycle. Details of validation of both pressure and flow measurements have been described previously [17]. The time taken to acquire both pressure and velocity data was approximately 5 minutes.

Carotid pressure and flow velocity data were sampled at a frequency of 200 Hz and digitised. After acquisition, waveforms were ensemble averaged off-line as previously described [17] using custom written software in Matlab (Mathworks, Natick, MA). Care was taken to ensure that only good quality beats (median 6 beats) were included in the ensemble. The members of the ensemble were identified by using the peak of the R wave as the fiducial point. After constructing the ensemble the members were checked for good temporal alignment. Occasionally, due to variability in the duration of isovolumic contraction period there was a small degree of misalignment (<5 ms) between the systolic rise phase of the beats and, if this was the case, any misalignment was corrected manually using the software. The cross correlation coefficient between the initial 600 ms of each beat was used as a quantitative measure of agreement between waveforms with a value >0.95 being regarded as acceptable. Local carotid artery pulse wave velocity, c, was calculated using the pressure-velocity loop method [17], [18]. Reproducibility of these methods has been previously published [17], [19] and the validity of the approach has been confirmed in vitro and in vivo [18], [20]. The within observer coefficient of variation was <10% for major waves.

### Augmentation Index, Waveform Type, Wave Intensity Analysis and Wave Separation

**Figure 1:**
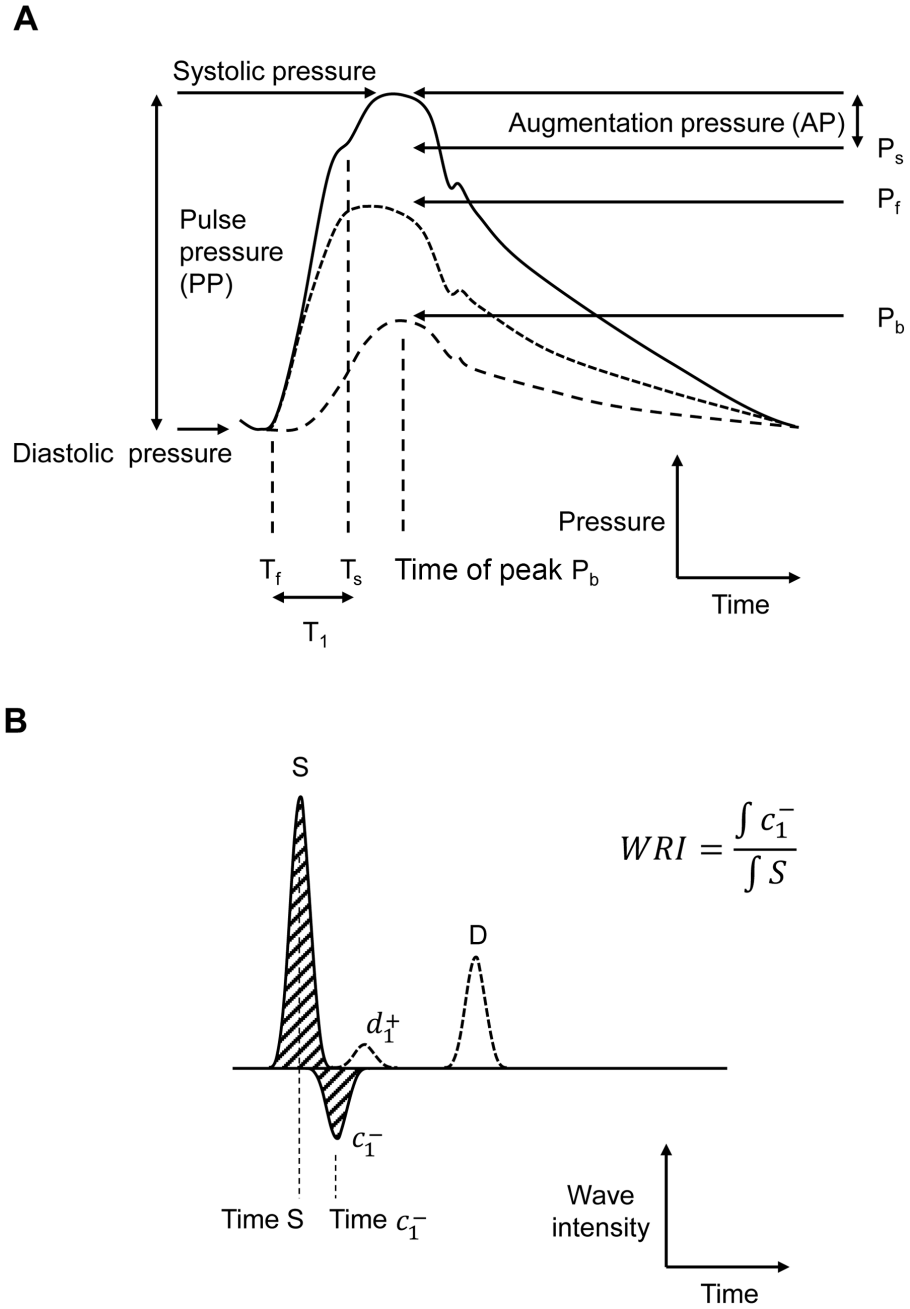
Examples illustrating definitions of measured parameters and indices. A) pressure waveform (blue) modified from [31] and indicating measured parameters and showing separated forward (black) and backward (red) components. Total pressure is the sum of forward and backward pressure. B) wave intensity analysis, showing principal waves and timings. Wave reflection index (WRI) is calculated as the ratio of the area under c^−^_1_ to the area under S wave. Abbreviations c^−^_1_, backwards (reflected) compression wave; D, forward decompression wave in late systole; d^+^_1_, mid-systolic forward decompression wave, presumed to be a re-reflection of the backward reflected compression wave, c^−^_1_; P_b_, peak backward pressure; P_f_, peak forward pressure; S, forward compression wave associated with ejection in early systole; T_f_, time of the foot of the pressure waveform; T_s_, time of the shoulder on the pressure waveform; T_1_, the time difference between foot and shoulder (T_s_−T_f_).

Augmentation index and the time of the shoulder (T_s_) were calculated from the pressure waveform as previously described [21] (figure 1). T_s_ was defined as the zero-crossing of the fourth derivative of the pressure and the timing of T_s_ was calculated with respect to the R wave on ECG to allow direct comparisons with the timing of other measures of reflection. The time difference between foot and shoulder (T_1_) was calculated (T_s_−T_f_) to allow comparison with other published data. Waves were also classified into 3 types as described previously [22]: Type A: patients whose peak systolic pressure occurred after the shoulder and AIx >12%, Type B: patients whose peak systolic pressure occurred after the shoulder, but 0< AIx <12% and Type C: patients whose peak systolic pressure preceded a shoulder and AIx <0.

Changes in pressure and flow in the circulation result from waves of varying magnitude, character and direction. The timing, magnitude, nature and direction of such waves can only be definitively established from combined pressure and flow data [2], [23]. Waves can originate either from the proximal (forward-travelling) or distal (backward-travelling) end of the circulation, and can be either compression or decompression waves (i.e. associated with a rise or fall in pressure). Wave intensity is a measure of the power density of a wave and is given by the product of the simultaneous incremental changes in local pressure (*dP*) and velocity (*dU*) in a given time interval [24]. The cumulative intensity of each wave (i.e. the integral under the wave) corresponds to the wave energy density (i.e. the work done by the wave).

Pressure changes due to forward-travelling (*dP_+_*) and backward-travelling (*dP_-_*) waves were separated using equations 1 & 2

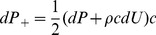
(1)

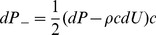
(2)where *ρ* is the density of blood (1050 kgm^−3^) and *c* is the carotid pulse wave velocity. This time domain approach to wave separation gives results that are essentially identical to frequency domain-based approaches [25]. Two measures of wave reflection were derived from pressure and flow data: the ratio of backward to forward pressure (P_b_/P_f_) [26], and wave reflection index (WRI), the ratio of the energy carried by reflected waves to the energy of the incident wave (S) due to left ventricular ejection [27] (Figure 1).

### Statistics

Statistical analysis was performed using Stata/IC (version 12.1, Stata Corp, College Station, TX). Continuous variables are reported as mean (standard deviation) or mean [95% confidence interval], categorical variables as n (%). Skewed data were log transformed. Correlations were assessed using Pearson’s correlation coefficient (r) or Spearman’s rank correlation coefficient (rho) as appropriate. Univariate and multivariate regression analysis was also performed. Interactions by gender were examined and included in models if p<0.05 for the interaction, otherwise the interaction term was dropped from the model and both genders were pooled.

## Results

The characteristics of the study participants are shown in table 1; women were shorter and lighter than men but body mass index, brachial BP or heart rate did not differ significantly.

**Table 1:** Characteristics of the individuals studied.

Measure	Total (n = 65)		Women (n = 22)		Men (n = 43)		p
Age, yrs	43.9	(14.1)	44.5	(15.8)	43.6	(13.3)	0.8
Weight, kg	74.6	(13.1)	65.2	(9.8)	79.9	(11.8)	<0.001
Height, m	1.73	(0.11)	1.65	(0.07)	1.78	(0.10)	<0.001
BMI, kg.m^−2^	24.80	(3.26)	24.01	(4.26)	25.26	(2.48)	0.2
SBP, mmHg	118.9	(10.6)	116.1	(9.9)	120.4	(10.8)	0.1
DBP, mmHg	72.3	(7.3)	71.7	(7.9)	72.6	(7.0)	0.6
HR, bpm	67.2	(10.3)	65.7	(8.0)	67.9	(11.3)	0.4
cSBP, mmHg	107.9	(10.4)	105.0	(10.5)	109.4	(10.2)	0.1
AIx, %	4.28	(16.13)	12.95	(14.95)	−0.26	(14.96)	<0.001
T_1_, ms	176.27	(55.71)	153.33	(52.99)	187.74	(54.03)	0.02
Type (A/B/C), n (%)	22/20/23	(34/31/35)	13/6/3	(59/27/14)	9/14/20	(21/33/47)	<0.001
P_b_/P_f_	0.13	(0.03)	0.12	(0.03)	0.13	(0.03)	0.4
Log WRI	−1.79	(0.42)	−1.82	(0.48)	−1.78	(0.40)	0.7

Data for men and women are also shown separately.

Data are mean (SD); p values were calculated using a Student’s t-test comparing women and men. AIx, augmentation index; BMI, body mass index; cSBP, central systolic pressure; DBP, diastolic blood pressure; HR, heart rate, Pb/Pf, the ratio of forward to backward pressure; SBP, systolic pressure; T_1_, the time difference between the foot and the shoulder of the waveform; WRI, wave reflection index.

### Interrelationships of Measures of Wave Reflection Magnitude and Timing

AIx did not correlate with WRI or P_b_/P_f_ (table 2), although AIx and log WRI correlated weakly when type C waveforms were excluded (i.e. AIx was restricted to positive values). In contrast log WRI and P_b_/P_f_ were closely correlated (table 2).

**Table 2:** Correlations between magnitude and timings of various indices of wave reflection.

Correlation	r	p
AI_x_ vs. log WRI	0.10	0.4
AI_x_ vs. P_b_/P_f_	−0.20	0.1
P_b_/P_f_ vs. log WRI	0.66	<0.001
AIx vs. log WRI (when AIx >0)	0.33	0.04
T_s_ vs. time of reflected wave	0.31	0.01
T_s_ vs. time P_b_	0.00	0.9
time c_1_^−^ vs. time of P_b_	0.82	<0.001
T_s_ vs. time of reflected wave (when AIx >0)	0.35	0.03

AIx, augmentation index; P_b_ backward pressure, P_b_/P_f_, the ratio of forward to backward pressure; T_s_, the time of the shoulder of the waveform; WRI, wave reflection index. Data are Pearson’s correlation coefficients.

There was a close positive correlation between the timing of c^−^_1_ and timing of P_b_, and a weaker but positive correlation between Ts and the timing of c^−^_1_ (table 2). There was no correlation between the timing of T_s_ and P_b._ There was a positive relationship between T_s_ and the time of c^−^_1_ when type C waveforms were excluded.

### Wave Patterns Associated with Waveform Types and their Relationship to Reflection and AIx

**Figure 2:**
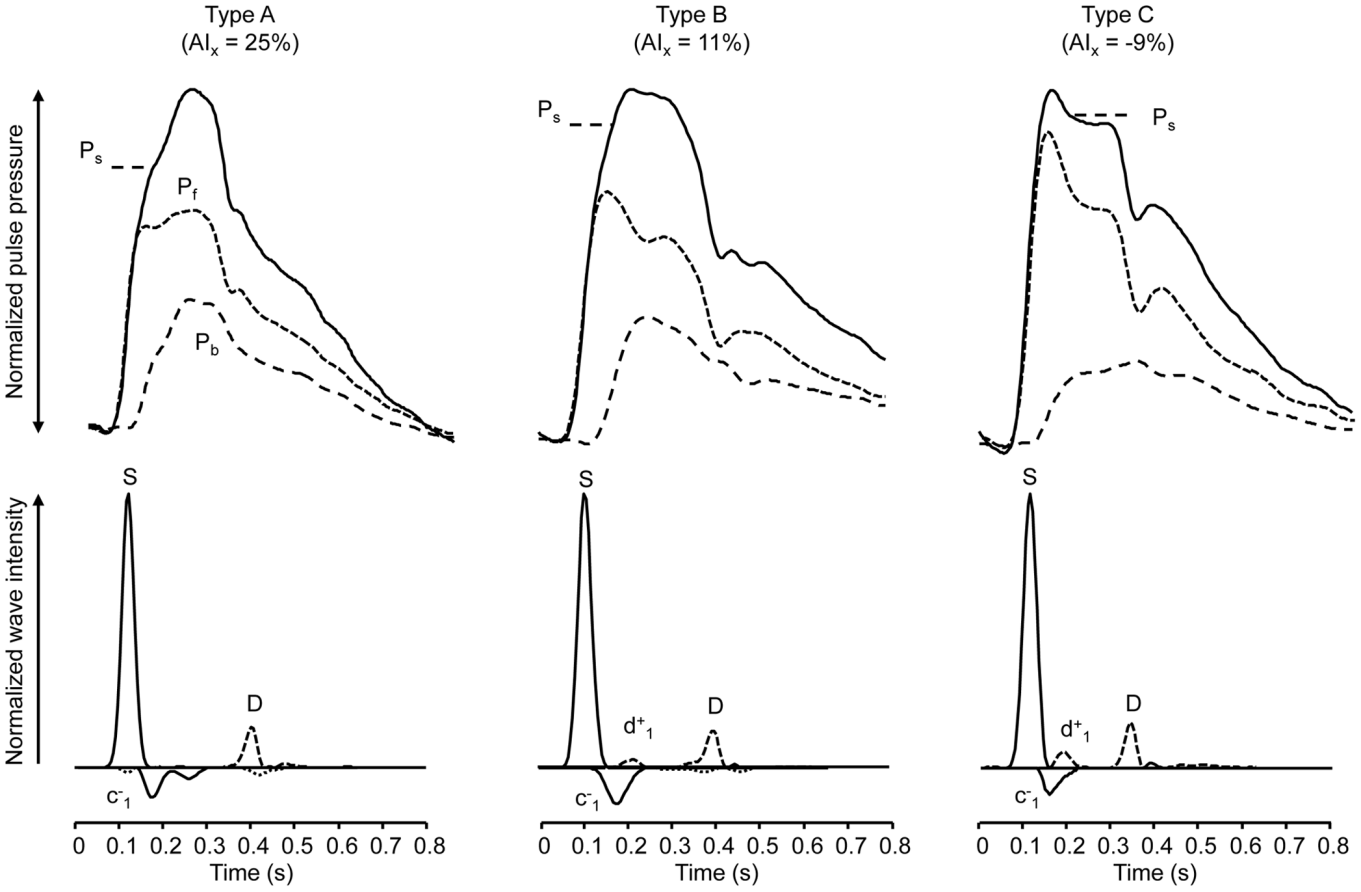
Wave intensity analysis and pressure separation of the 3 different types of pressure waveform. The three types of pressure waveform (A, B, C) and their respective augmentation indices (AIx) are shown. The magnitude of the pressure and wave intensity traces have been scaled equally to allow comparison of morphology. Three principal wave S, c^−^_1_ and D, forward pressure (P_f_) backward pressure (P_b_) and the shoulder point (P_s_) are indicated.

Three types of waveform (A, B & C) were seen in the carotid artery (Figure 2). Wave intensity analysis showed a typical pattern of waves in the carotid arteries with a large forward compression wave (S) associated with ejection, followed by a backward (reflected) compression wave (c^−^_1_) and a forward decompression wave in late systole (D), prior to closure of the aortic valve. Another forward decompression wave (d^+^_1_) was seen frequently in mid-systole, but its magnitude varied considerably between different types of wave form. Negative augmentation in the carotid artery (as typified by the Type C wave shown in figure 2) was attributable to a large decompression wave (d^+^_1_) that was associated with a fall in both pressure and flow resulting in the decline in peak pressure responsible for the negative AIx. There was a strong negative correlation between the energy carried by d^+^_1_ and AIx (Spearman’s rho = −0.69; p<0.001) suggesting that the magnitude of AIx in type C waveforms is largely determined by this re-reflected decompression wave. There was no difference between measures of wave reflection (log WRI or P_b_/P_f_) between type A, B and C waveforms (Table 3).

**Table 3:** Comparison of measures of reflection between type A, B and C waveforms.

	Type A (n = 22)	Type B (n = 20)	Type C (n = 23)	p
log WRI	−1.74 (−2.89, 0.81)	−1.87 (−2.77, 1.00)	−1.79 (−2.49, 1.01)	>0.9
P_b_/P_f_	0.12 (0.06, 0.19)	0.12 (0.07, 0.16)	0.14 (0.09, 0.21)	0.1

Data are mean (95% confidence intervals). P values were calculated by analysis of variance.

### Relationships between Indices of Wave Reflection and Age, Height and Sex

**Figure 3:**
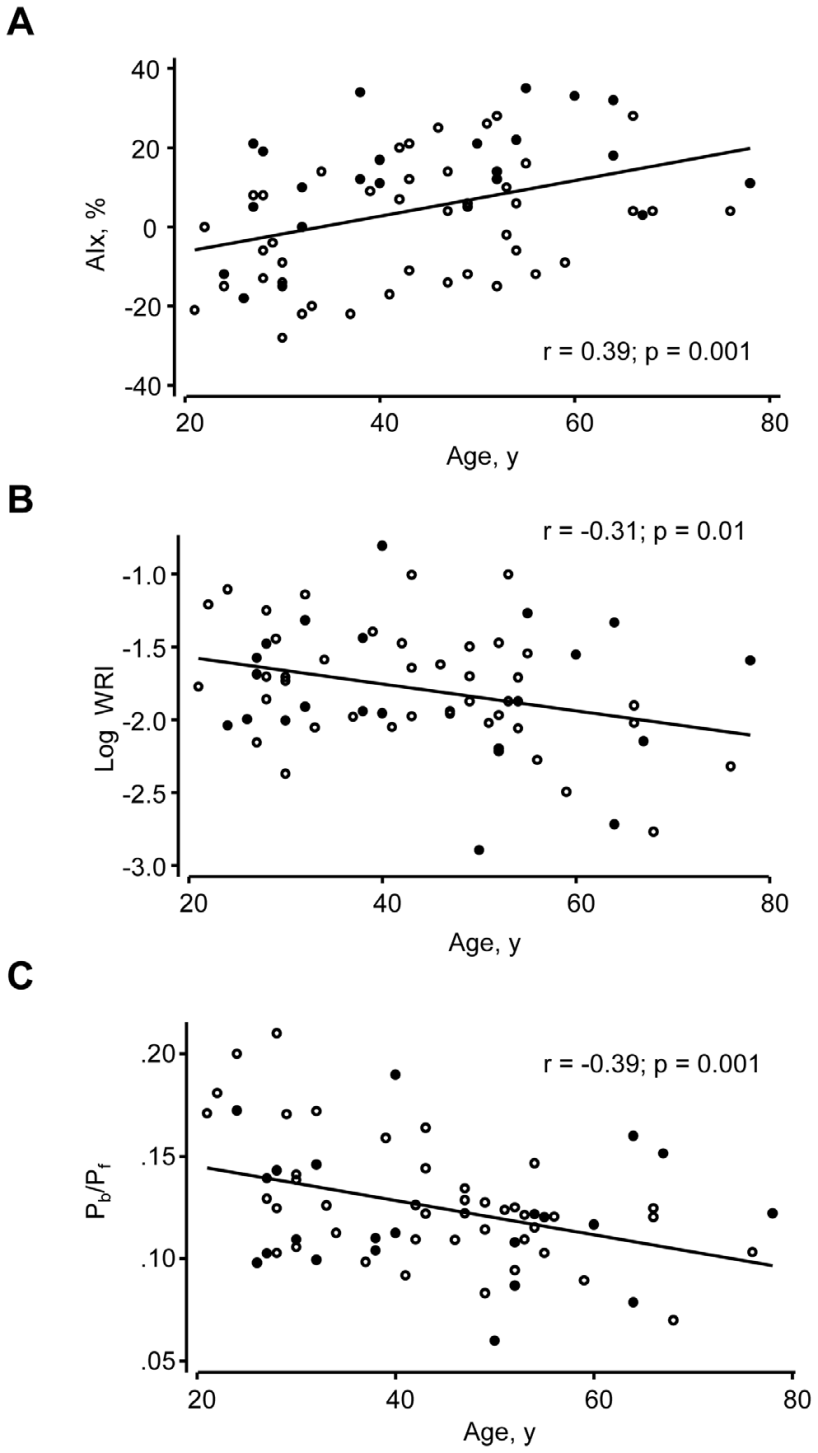
Scatterplots of the relationship between age and various indices. A) Age vs. AIx B) Age vs, Log wave reflection index (WRI) and C) Age vs. peak backward/peak forward pressure (P_b_/P_f_). Regression lines are derived from data pooled by gender but data points for men (○) and women (•) are indicated separately.

**Figure 4:**
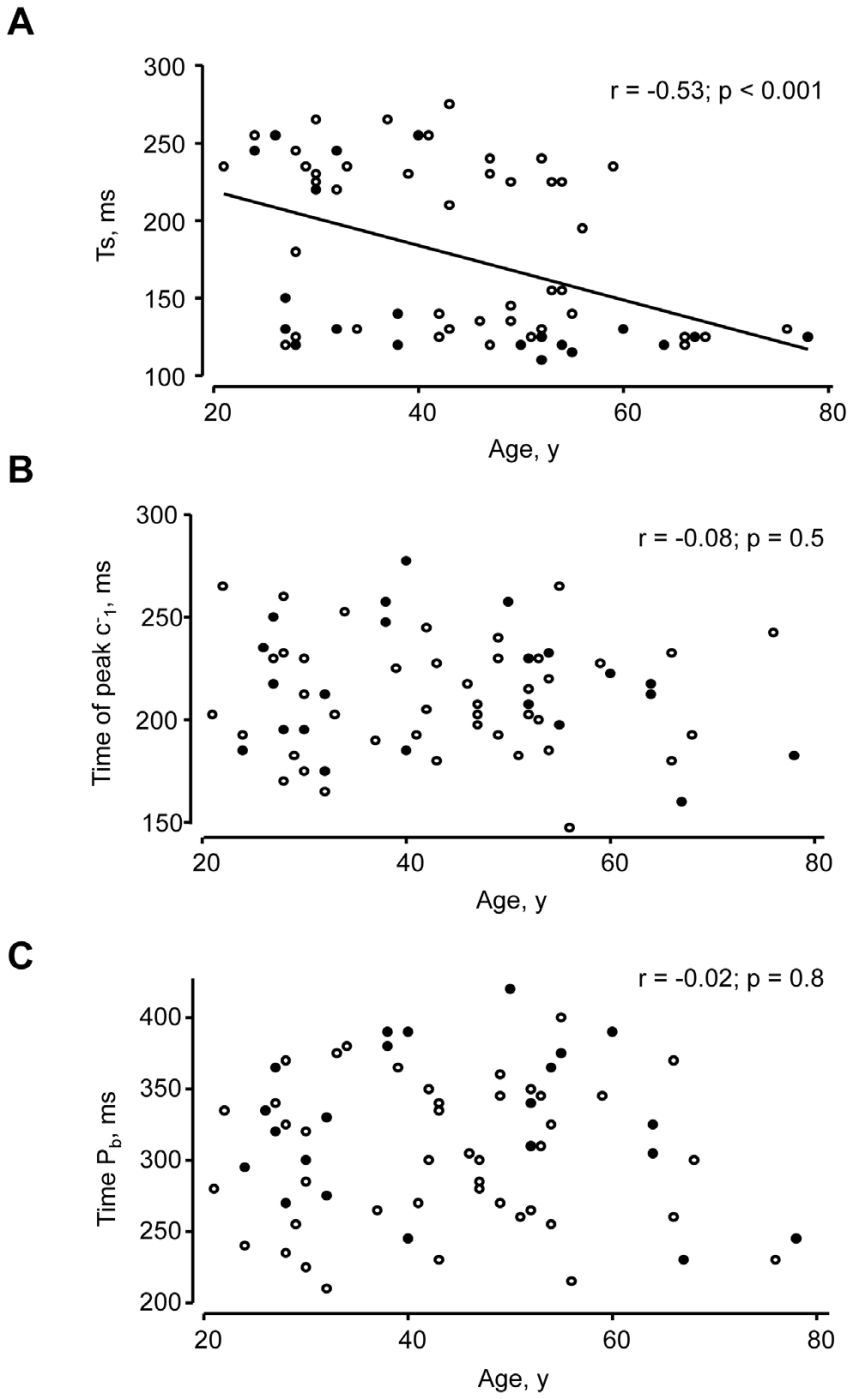
Scatterplots of the relationship between age and wave or waveform timings. A) time of the shoulder (Ts), B) time of the peak of the reflected wave, c^−^_1_ and C) time of P_b_. Regression lines are derived from data pooled by gender but data points for men (○) and women (•) are indicated separately.

There was a significant positive relationship between age and AIx (r = 0.39; p = 0.01; figure 3A). In contrast, there was a negative linear relationship between log WRI and age (r = −0.31; p = 0.01; Figure 3B) and between P_b_/P_f_ and age (r = −0.39; p = 0.001; Figure 3C). If type C waveforms were excluded there was no longer a significant correlation between AIx and age (r = 0.14; p = 0.4). There was a negative relationship between T_s_ and age (figure 4A), but there was no significant relationship between the time of the reflected wave, c^−^_1_ and age (Figure 4B) or time of the peak backward pressure (time P_b_) and age (Figure 4C).

There was a highly significant inverse association between height and AIx but there were no significant associations between height and log WRI or height and P_b_/P_f_ (table 4). T_s_ was positively correlated with height but there were no significant associations between height and time of c_1_^−^ or height and time of P_b_.

**Table 4:** Correlations between magnitude and timings of various indices of wave reflection and height.

Correlation	r	p
AIx vs. height	−0.43	0.001
log WRI vs. height	0.20	0.1
P_b_/P_f_ vs. height	0.17	0.2
T_s_ vs. height	0.52	<0.001
Time of reflected wave vs. height	0.26	0.06
Time of P_b_ vs. height	−0.15	0.3

AIx, augmentation index; P_b_ backward pressure; P_b_/P_f_, the ratio of forward to backward pressure; T_s_, the time of the shoulder of the waveform; WRI, wave reflection index. Data are Pearson’s correlation coefficients. Data are Pearson’s correlation coefficients.

AIx was higher in women than men (13.0 (6.3, 19.6)% vs. 0.2 (−4.5, 4.8)%; p<0.001), whereas log WRI (−1.82 (−2.03, −1.60) vs. −1.78 (−1.90, −1.65; p = 0.7) and P_b_/P_f_ (0.12 (0.11, 0.13) vs. 0.13 (0.12, 0.14); p = 0.4) did not differ by sex. T_s_ was also earlier in women than men (211 (184, 239)ms vs. 249 (230, 268)ms; p = 0.02) but neither time of c_1_^−^ (216 (202, 230)ms vs. 211 (203, 220)ms; p = 0.5) nor time of P_b_ (327 (304, 351)ms vs. 302 (287, 318)ms; p = 0.1) differed by gender.

In this group of healthy normotensive individuals there was only a weak relationship between brachial systolic BP and age (r = 0.11; p = 0.4) but there was a highly significant positive correlation between age and carotid pulse wave velocity (r = 0.39; p<0.001).

## Discussion

This study has compared the relationship between AIx and measures of wave reflection derived from wave intensity and wave separation across the adult age range in healthy men and women. The ‘gold standard’ measures of wave reflection calculated from pressure and flow data gave results that were consistent with one another, but did not correlate with AIx, except for a limited degree of correlation when type C waves were excluded. Similarly there was poor agreement between timings of wave reflection derived from pressure waveform analysis (T_s_) compared with those based on pressure and flow data. Wave intensity analysis showed that the characteristic morphology of type C waves is due to a forward travelling decompression wave in mid-systole and that use of the shoulder as an indication of timing and magnitude of reflection is inappropriate in this type of waveform. Use of AIx as a measure of wave reflection was shown to give misleading results in terms of the relationships with age, height or gender, when type C waves were included in analyses.

Several studies have examined the relationship between AIx and age, height and gender [9]. Our observations are consistent with published data showing that older age is associated with a rise in AIx and a decline in T_1_; that height is inversely associated with AIx; and that AIx is higher and T_1_ is lower in women [11]–[14]. Only a limited number of previous studies have used pressure and flow velocity data to assess wave reflection in humans. The Asklepios study [12] of people aged between 35–55 yrs saw a much less marked increase in P_b_/P_f_ than AIx with increasing age and reported no difference in P_b_/P_f_ between men and women. This study also found only modest agreement between AIx and P_b_/P_f_ and further analysis of these data showed that the time of the shoulder did not correspond with the time of arrival of the reflected wave [28]. A study of participants in the Framingham Offspring and Third Generation study [3] reported that P_b_/P_f_ rose slightly with age up to approximately 50 years and then declined. It is noteworthy that both these studies did not exclude people with high blood pressure. Given that indices of reflection increase with increasing BP [3] it seems plausible that some differences between these studies and ours are attributable to our exclusion of people with hypertension, but taken together these studies indicate serious limitations to AIx as a measure of wave reflection.

Our study provides new hemodynamic insights into why AIx does not agree closely with other measures of wave reflection, particularly in the case of type C waveforms. Use of AIx as a measure of reflection is complicated by several factors including the influence of pulse wave velocity, left ventricular ejection patterns, difficulties in identifying a shoulder corresponding to the time of arrival of the reflected wave when it occurs early in systole. In the case of type C waveforms problems of interpretation are further confounded by the presence of a forward travelling decompression wave that causes a late shoulder in the pressure waveform and gives rise to negative values of AIx. The mechanism accounting for the forward decompression wave in mid-systole remains to be fully established, but it is also prominent in the brachial and radial artery of normal individuals [17] and is likely to be due to re-reflection of the backward travelling reflected wave. As it returns toward the heart, the reflected wave in the carotid artery will encounter an impedance mismatch due to the marked increase in cross-sectional area at the origin of the common carotid artery or brachiocephalic artery and consequently undergo re-reflection as a decompression wave. A similar suggestion has been made based on numerical modelling studies of wave reflection in the upper arm [29]. We conclude that negative values of AIx should not be interpreted as ‘negative’ wave reflection (or included in correlation or regression analyses assessing wave reflection where such an interpretation is implicit). If measurement of AIx is restricted to type A and B waveforms then it appears to give some limited insight into wave reflection. Nevertheless even when type C waves are excluded the correlation between AIx and more accurate measures of wave reflection is, at best, modest and interpretation of positive AIx as a measure of wave reflection should be made with caution.

Our study has several limitations. We chose to recruit participants without hypertension or evidence of cardiovascular disease. This has the advantage that our observations are uncomplicated by presence of disease or effect of therapy but has the disadvantage that this sample is not representative of the general population, particularly in terms of BP. Current data indicate that ∼41% and 70% of people in US between 45–65 and over 65 years respectively have hypertension (defined as a systolic BP≥140 mm Hg, a diastolic BP≥90 mm Hg, or taking high blood pressure medication) [30]. Exclusion of people with hypertension is likely to have led to our sample being a ‘super’ healthy population particular at older ages. Despite this there was a clear positive relationship between carotid pulse wave velocity and age, suggesting that the sample is not unrepresentative, at least in respect of vascular aging. We studied a relatively small number of healthy individuals and while most relationships appeared linear the study has limited power to detect non-linear relationships. Measurements in this study were made in the carotid artery rather than the aorta. However carotid and aortic AIx are very closely correlated [11] and comparison of AIx with other ‘gold standard’ measures of wave reflection at the same site is the most appropriate comparison, even if the extent of wave reflection is not necessarily identical to that in the aorta.

In conclusion these data indicate that AIx has major limitations as a measure of wave reflection: this is particularly the case for type C waveforms (i.e. when AIx is negative). Type C waveforms are relatively common in younger individuals and men and the inclusion of negative AIx values in analyses will distort relationships between wave reflection and aging, height or gender. We propose that if AIx is to be used as a crude index of wave reflection then type C waves (negative values of AIx) should be excluded from analyses.
